# Factors that impact access to ongoing health care for First Nation children with a chronic condition

**DOI:** 10.1186/s12913-018-3263-y

**Published:** 2018-06-14

**Authors:** Julieann Coombes, Kate Hunter, Tamara Mackean, Andrew J. A. Holland, Elizabeth Sullivan, Rebecca Ivers

**Affiliations:** 1 The George Institute for Global Health, Level 5, 1 King St, Newtown, NSW 2042 Australia; 2 University of Technology Sydney, 15 Broadway St, Ultimo, NSW 2007 Australia; 3 Flinders University, Adelaide, SA Australia; 4 The Children’s Hospital, Westmead, Cnr Hawkesbury Rd and Hainsworth St, Westmead, NSW 2145 Australia; 5 Faculty of Medicine, UNSW, Sydney, Australia; 6 Sydney Medical School, University of Sydney, Sydney, Australia

**Keywords:** Children, First Nation, Chronic condition, Healthcare, Access

## Abstract

**Background:**

Access to multidisciplinary health care services for First Nation children with a chronic condition is critical for the child’s health and well-being, but disparities and inequality in health care systems have been almost impossible to eradicate for First Nation people globally. The objective of this review is to identify the factors that impact access and ongoing care for First Nation children globally with a chronic condition.

**Methods:**

An extensive systematic search was conducted of nine electronic databases to identify primary studies that explored factors affecting access to ongoing services for First Nation children with a chronic disease or injury. Due to the heterogeneity of included studies the Mixed Method Appraisal Tool (MMAT) was used to assess study quality.

**Results:**

A total of six studies from Australia, New Zealand and Canada were identified and included in this review. Four studies applied qualitative approaches using in-depth semi structured interviews, focus groups and community fora. Two of the six studies used quantitative approaches. Facilitators included the utilisation of First Nation liaison workers or First Nation Health workers. Key barriers that emerged included lack of culturally appropriate health care, distance, language and cultural barriers, racism, the lack of incorporation of First Nation workers in services, financial difficulties and transport issues.

**Conclusion:**

There are few studies that have identified positive factors that facilitate access to health care for First Nation children. There is an urgent need to develop programs and processes to facilitate access to appropriate health care that are inclusive of the cultural needs of First Nation children.

## Background

Shared histories of colonisation and disempowerment have had a devastating impact on health and social outcomes for First Nations peoples globally [1–4]. First Nations people still struggle for survival, equality and equity in health, education, housing, employment and the right to good health and wellbeing [3]. The effect of Homelands being taken away, child removal, cultural and social dislocation together with the combined losses of culture, moiety systems and loss of language, contribute to marginalisation from services such as health, education and child and family services. This in turn can lead to a loss of self-determination [5] and other adverse outcomes, such as alcohol and substance abuse, domestic violence, poverty, poor health outcomes and higher mortality rates, high out-of-home care cases, high rates of incarceration and suicide [6, 7]. These accumulated difficulties have had a significant impact on the health and well-being of First Nation children and their children’s children for many generations [2, 8].

As a result, First Nation children globally are overrepresented in chronic conditions and injuries [9–12], including renal disease [13], respiratory diseases [14], rheumatic fever, diabetes [7], skin diseases [15], unintentional injury [16, 17] and burns [18–20].

Optimal recovery for such conditions is associated with ongoing access to culturally appropriate multi-disciplinary health services and health promotion resources [21]. However, disparities in terms of access to services delivered and outcomes for First Nation children remain [3, 11]. Despite significant overrepresentation of First Nation children with long term chronic conditions including those because of an injury, little is known about factors that impact access to required ongoing health care once discharged from a tertiary health center. A systematic review was carried out to examine factors (facilitators and barriers) that impact access to ongoing health care for First Nation children with a chronic health condition or injury.

## Methods

### Inclusion criteria

This literature review included global research studies published in English from 2000 to 2017. Publications were included if they reported primary research focusing on First Nation children, aged 0–16 years with a chronic condition, and their access to health care. Both quantitative and qualitative research designs were included. (Table 1 shows search terms used).

**Table 1:** Search terms used to identify relevant studies

	Age
1	Child*
2	Paediatric
3	Infant
	Population
4	Indigenous
5	Aborig*
6	Torres Strait Islander
7	Inuit
8	First Nation
9	Maori
10	Native American
11	Native
12	Sami
	Condition
13	Injury
14	Chronic condition*
15	Long-term conditions
16	Illness
17	Complex conditions
18	Injuries
19	Wounds
	Health care
20	Health Care
21	Disparities
22	Community health services

*Indicates truncated word

To locate relevant studies two methods were used: (a) a search of data bases for primary papers from AIATSIS (Indigenous Studies Bibliography), CINAHL (The Cumulative Index to Nursing and Allied Health Literature), Rural and Remote Health Database, ATSI (Aboriginal and Torres strait Islander) Health Informit Online, Web of Science, Medline, PubMed, Cochrane Library and Australia Indigenous HealthInfoNet. (b) A hand search of references from identified studies (Table 2).

**Table 2:** Databases searched by date and the number of possible relevant records corresponding to search terms

Date	Database	Number of records
25/03/2016	AIATSIS: Indigenous Studies Bibliography	0
25/03/2016	ATSIhealth	0
25/03/2016	Medline	24
28/03/2016	CINAHL	1
28/03/2016	Ruraul Data Base	0
28/03/2016	Health Info Net	0
29/03/2016	Lowitja	1
29/03/2016	Cochrane Library	4
29/03/2016	INFORMIT	1

### Assessment of included papers

Papers were assessed for potential inclusion based on the abstract and title. For those papers that seemed appropriate the full text was accessed and any duplicates removed. Full text papers were judged against the inclusion criteria. Included papers were summarised using a qualitative synthesis and were independently reviewed by two authors (JC and KH) with a unanimous agreement as to which papers were to be included. The quality of included studies was assessed using the Mixed Methods Appraisal Tool (MMAT) [22] (see Table 3 for details). The MMAT has previously been shown to be a comprehensive tool for assessing mixed method studies and meets the accepted standards for validity and reliability [23, 24]. The MMAT tool has criteria for each study type and assigns an overall percentage ranking.

**Table 3:** Assessment of included papers (MMAT)

#	Author Date Country	Aims	Methods	Participants and setting	Analysis	Key findings	MMAT Scores
1	Michelle DiGiacomo 2013 Australia	To identify factors involved in accessing services and support for Aboriginal children with a disability	Community fora	Group 1: Parents and carers of Aboriginal children with a disability (5) Group 2: Health and social Service providers (17) Setting: ACCHS in metropolitan Sydney;	Framework analysis – consensus with co-authors and community members.	Both groups: Lack of awareness of services; Inadequate availability of services Carers: Racism; Insufficient or non-existent services; Providers: Logistical barriers; Cultural and historical issues impacting on effectiveness of services Suggested solutions: Need for an enhanced role of ACCHS and AHWs dedicated to support children with disability; school-based support; routinely updated information; Inter-sectoral partnerships	****
2	Rob Watson 2012 Unama’ki (Cape Breton), Nova Scotia, Canada	To Identify gaps in Asthma Education, Health promotion and Social Support for	Community based Participatory research	Group 1: Mi’kmaq parents and carers of youth with asthma Setting 1: Mi’Kmag communities Group 2: Mi’Kmag youth aged 8–12 yrs. diagnosed with asthma Setting 2: A 2-day camp*.*	All data collected from study was analysed by thematic framework	Both groups: There is a lack of support in the areas of social, educational and culturally appropriate resources. Barriers in accessing availability of services Suggested solutions: The need for involvement and collaboration with First Nation people for culturally appropriate support and educational resources for asthma education and intervention.	****
3	Shanthi Ameratunga 2010 New Zealand	To identify key issues and barriers to ongoing health care following hospitalisation for children who were admitted to hospital following unintentional injury:	A qualitative research design using Interviews and focus group	Group: In-depth individual interviews and 3 focus groups with 21 service providers’ and families of children hospitalised with an injury. Setting: Health service departments.	Interviews were transcribed and data was analysed using a thematic framework	Key issues agreed on by both service providers and participating families included the inabilities to meet the needs of the children’s emotional needs, lack of family support, lack of culturally appropriate resourses, poor coordination of hospital and community health services and lack of aftercare follow ups. Suggested solutions: Ensuring culturally appropriate services are available and the need for cultural competency for service providers. Interventions to improve services at provider and patient levels, improvement in the development of clear concise discharge plans and the need to support families	***
4	S.L. Thomas 2015 New South Wales, Australia	Investigating how to improve partnerships with services and First nation families to maximize better health outcomes for First Nation children.	Semi-structured interviews and Focus Groups	Group 1: Focus Groups with community-based service providers. Group 2: Semi-structured interviews with service managers	Views of participants were documented and a thematic analysis was then used.	There is a need to improve paediatric outreach services for urban First Nation children through leadership, partnerships and culturally appropriate child health care based on a holistic model of care. Suggested solutions: Collaboration between health services and community members will improve access to services for First Nation children.	**
5	R.Cresp 2016 Western Australia	To determine whether a culturally appropriate program could improve attendance to out of hospital appointments	Quantitative evaluation	First Nation children aged 0–19 yrs. who resided in Kimberley, Pibara or Perth regions	Pre-post de-identified data on hospital admissions, length of stay, emergency department presentations and outpatient appointments was used for the analysis	Findings suggest it is health outcomes for l children by engaging families with health services, improving communication between health service providers, and coordinating First Nation service provider led care.	****
6	R.Eley 2010 Victoria Australia	To give young First Nation people a knowledge and understanding of asthma and greater management over their asthma through a culturally safe and enjoyable process.	Quantitative measurement using medical reviews and spirometry and qualitive research using community based participatory research	First nation young people aged between 5 and 17 yrs.	Study 1. Analysis of respiratory data were compared at different time intervals. Study 2. Interviews of participants were documented and a thematic analysis was also used	Study 1. There was an improvement in respiratory function. Study 2. The participants knowledge of asthma increased, asthma action plans were developed and there was greater compliance with medication. Other health benefits also achieved included the removal of barriers to accessing further medical services.	***

**MMAT Score 25-50%, ***MMAT Score 50-75%, ****MMAT Score 75-100%

This paper is reported in accordance with the PRISMA (preferred reporting items for systematic reviews and meta-analysis) reporting guidelines provided for systematic reviews and meta-analyses [25].

### Ethics

All data were extracted from published manuscripts and therefore did not need ethics approval.

## Results

**Fig. 1:**
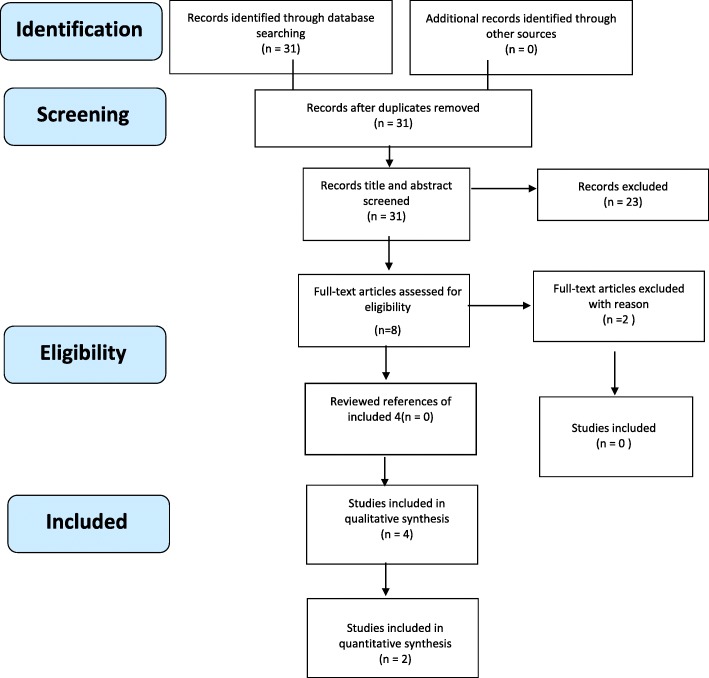
Prisma Flow Chart

The electronic database search returned 31 relevant records and no additional records were identified by a manual search of references. After assessing the records for relevance 11 reference citations were saved and full text was obtained and reviewed for relevance to the research questions. From these articles, 6 met the inclusion criteria for the review. Of the six included studies, four studies were based in Australia [26–29], one from New Zealand [16], and one from Canada [30] (See fig. 1 for results).

### Included study methods and aims

For this review the definition of a chronic condition in children is a disease or illness that occurs in children between 0 and 16 yrs. and has been present for more than 3 months or if the disease or illness has occurred more than 3 times in the past year, and includes major trauma caused from an injury [31, 32].

Four included studies reported results from qualitative research. Methods included the conduct of focus groups by Thomas et al. [27], interviews by Ameratunga et al. [16], the conduct of community fora by DiGiacomo et al. [26] and Watson et al. [30] incorporated a community based participatory research approach. One Australian study held focus groups in a Community Controlled Health Service to establish factors involved in accessing services for Australia’s First Nation children with a disability [26]. The second study from Australia used semi-structured interviews and focus groups with community based child health services and Service Managers to investigate the importance of partnership with First Nation families and health services [27]. The third study from Australia was a quantitative study by Cresp et al. [28] which focused on pre-post de-identified data on hospital admissions, length of stay, emergency department presentations and outpatient appointments. The fourth study by Eley et al. [14] from Australia included qualitative and quantitative methods.

A study from New Zealand by Ameratunga et al. [16] conducted interviews and focus groups to identify key issues and barriers to ongoing health care following hospitalisation for children who sustain an injury. A community based study from Canada conducted by Watson et al. [30] examined the support and educational needs preferred by children with asthma by collecting data through participant observation, sharing circles and focus groups; these were conducted at a 2 day camp for children and carers. From these six studies, none mention the use of Indigenous research.

### Quality of studies

The quality of included studies varied. Of the 4 studies one had a MMAT score of ** (50%) [27], one study was scored at ***(75%) [16] and two scored ****(100%). The two highest ranked studies included participatory research [30] with expressions from First Nation people as well as service providers [26]. Using the MMAT tool resulted in an overall methodology score which was then calculated into a percentage [23].

### Facilitators to accessing health care

#### First nation health work force

Three studies from Australia presented evidence on how crucial the work of Aboriginal Health Workers is in partnership with community health services, in creating and maintaining culturally competent healthcare systems for First Nation children and their families [26–28]. Aboriginal Health Workers and Aboriginal Liaison Workers (hereafter referred to as AHW and ALW) roles are diverse and some of their roles include, but are not limited to, communicating and facilitating access to other services, providing care that meets the social, physical, emotional and cultural needs to achieve better health outcomes for First Nation patients.

Another Australian study reported that non-attendance at appointments decreased among children who were involved in the Koorliny Moort program which consisted of a First Nation senior program manager, a First Nation senior social worker, two First Nation Liaison Officer (IRR, 0.83; 95% CI, 0.74e0.94; *P* < 0.001) [28]. AHWs helped cultivate a trusting environment for First Nation children and families, ensuring that First Nation peoples’ holistic concepts of health and healing were respected [27].

#### Community engagement

Studies from Australia also describe the importance of community engagement in accessing health services. It was reported that primary health community services working with AHW’s played an integral role in cultivating a trusting environment helping to overcome cultural barriers [28].

These studies also found that culturally appropriate child health services which collaborated with First Nation community organisations [27, 28], First Nation families [27, 28], First Nation Elders [29] and AHW who are local to that community [29] saw improved access to appropriate services [27, 29]. Another study found that cultivating relationships with communities created a culturally safe environment and reported families were more likely to drop-in for health care [27].

### Barriers to accessing health care

#### Transport and finance

Geographical locations due to remoteness and distance to services made it difficult for parents to bring their child in for appointments. Vehicles were not available to families due to low finance and public transport costs became a burden after multiple visits to clinics or other health care facilities [26].

#### Cultural competency

Lack of cultural competency in the service was also described as a barrier by Thomas et al. [27], she describes the importance of implementing a culturally appropriate model of care based on trust and respect [16, 26, 30].

#### Language

Studies reported barriers due to language and promotional material not being culturally appropriate [26, 30]. The lack of interpreters was another reason why parents did not attend health care services with their children [16].

#### Coordination

In Aotearoa, New Zealand a qualitative research study was conducted using in-depth individual interviews and focus groups with services providers who were involved with the care and support of First Nation children who had sustained an unintentional injury. A lack of coordination between hospital and community services were identified as barriers [16].

#### Adequate follow up plans

Several studies reported the need for a clear, well developed follow-up plan, and that support systems for ongoing health care was imperative for First Nation children once they were discharged from the hospital to ensure best quality ongoing healthcare [16, 28].

#### Fear of child removal

Long held beliefs and racism have a lingering impact on how health workers care for First Nation children and their families, with child protection services often being contacted [16, 26]. This has cemented the fear of children being taken from families when attending health care services.

#### Staff turnover

High staff turnover has been globally problematic with medical and nursing staff seeing the child just once during the child’s ongoing medical appointments. This leads to fractures in the continuity of healthcare [27].

## Discussion

First Nation children have a basic human right to the best possible culturally safe health care [8, 33]. There have been many studies and research papers written about the burden of chronic conditions and injury in First Nation children [13, 17, 18, 30, 33–38]. This review has found there is little previous work that addresses the factors that impact access to ongoing health care for First Nation children globally who have a chronic condition [28]. Western research is inevitably going to influence the interpretation of data from the non- Indigenous researcher due to imperial and colonial discourse [1]. The included papers in this systematic review were written by researchers who have not mentioned the development of indigenous research agendas, methodologies or protocols.

Being able to readily access appropriate health care is an essential aspect for all chronic conditions, but it is unclear how well this occurs for First Nation children. Ensuring that ongoing care is planned, culturally appropriate and inclusive of First Nation children and their family is paramount to maintaining and improving access.

The accessibility of high quality and effective ongoing care for First Nation children, who constitute a high proportion of patients with a chronic condition or injury, particularly those from regional and remote settings in Australia, New Zealand and Canada is particularly important given the complexity of their need for long term healthcare [39]. Access to a holistic health care system that is culturally appropriate and culturally sensitive is essential to produce good long term health and wellbeing outcomes [27, 40]. However, there is little evidence around what is most effective and most importantly what can facilitate that access. Notably, there were no studies identified for First Nations peoples of the United States, suggesting significant research gaps exist [34, 41]. This review builds on what is known about barriers to accessing acute health care such as the transgenerational fears from past treatment of First Nation people due to institutionalised racism from health service, the parent’s fear of their child being removed from home by the welfare system and the lack of cultural sensitivity and awareness in health care services [7, 8].

From this review, we also identified positive factors that facilitate access to health care for First Nation people. Studies have shown that First Nation health/liaison workers employed in health settings appear to alleviate some of the previously mentioned barriers and can be beneficial to better health outcomes [10, 28, 42] yet the lack of funding and resources in health institutions impedes on the hiring of First Nation staff [9, 16, 36]. There was clear acknowledgement of the need for First Nation health/liaison workers within health care centers. First Nation health workers have become instrumental in providing culturally appropriate health care and support for First Nation people in hospital settings and private clinics globally [42], which helps alleviate the impact of racism in such settings. Being able to talk to a health worker who understands culture improves a patient’s understanding of the care and treatment needed for improved health outcomes. This in turn can reduce fear, mistrust and racism [28].

In this review we found that having culturally competent health facilities was effective for First Nation people, and this is also evident from other studies examining other First Nations child health programs. In Western Australia, the Koorliny Moort program was designed specifically for Australia’s First Nation children and also demonstrated the benefits of integrating First Nation health workers by engaging families with health services, improving communications and improved access to out of hospital health care [28]. There is also evidence that this approach is important for other marginalised populations.

One such example is a pilot study conducted by Nordin Dahhan et al. in Amsterdam describing an approach called *The Mosaic Outpatient Clinic* [10] (hereafter referred to as MOC). The MOC was set up in outpatient clinics at 3 hospitals in Amsterdam, specifically for Ethnic minority children who experienced chronic health issues such as diabetes type 1, asthma and/or a metabolic disease. The aim of the MOC was to provide an insight into what bottlenecks prevent access to health services and better health outcomes for ethnic minority children who are socially disadvantaged. Although this study did not include First Nation children it did show that integrating student healthcare workers as cultural mediators that served at the patient-clinician interface to translate language and interpret cultural differences were identified as beneficial. Results of MOC trial showed that patients and families were more appreciative of services and had a greater insight to the child’s chronic illness when the participants had access to a cultural mediator [10]. In addition, this review found that families and care providers reported the lack of culturally appropriate health information materials impacted their access to services. Such materials could be seen as interesting, informative and can improve quality of life [30]. Community produced health messages with designs and images that reflect their community’s culture would be more appealing to engage with. Involving community members in the development would also contribute to the cultural relevance of health related material [21, 30].

## Conclusion

First Nation people are one of the most researched populations in the world [43]. Despite this, the existing health disparities and unavailability of culturally appropriate and inclusive health care services are still evident today [8, 12]. Studies of First Nation children with a chronic condition, have typically been conducted using Western paradigms based on Western philosophies and western concepts of health [1, 44, 45]. As claimed by First Nation researchers in Australia and New Zealand the use of a western framework to interpret Indigenous knowledge is oppressive. Western frameworks are limiting because they often fail to capture the complexities of indigenous ways of knowing, potentially simplifying important cultural concepts and constructs [46].Although this review found few studies examining factors impacting on access to ongoing health care for First Nation children, several themes emerged from the studies that were identified. These included barriers such as racism in health services, linguistic, lack of cultural appropriate health promotional material, and lack of cultural understanding from service providers. In conclusion, this review demonstrates that by understanding diversity and cultural background, service providers can deliver culturally competent care within Health services. Also, appropriate levels of funding to support culturally safe healthcare services that support Aboriginal Health Workers, Aboriginal Liaison Workers, transport and finance will assist in ensuring ongoing access for First Nation children with a chronic condition.

### Strengths and limitations

There is difficulty in ascertaining the exact factors that impact access to ongoing health care for First Nation children globally due to the limited previous research. There are many limitations and biases to the evidence that currently exists such as cultural appropriateness of the studies and also the use of western methodology versus the use of Indigenous methodology and the different views of First Nation concept of health and well-being to Western views of health [1, 3, 44, 45].

A strength of this systematic review was that it was led by a First Nation researcher, ensuring the included studies were viewed through the lens of a First Nation perspective using an interface method [47].

## Abbreviations

AHW: Aboriginal Health Workers
AIATSIS: Australian Institute of Aboriginal and Torres Strait Islander Studies
ALW: Aboriginal Liaison Workers
ATSI: Aboriginal and Torres strait Islander
CINAHL: Cumulative Index to Nursing and Allied Health Literature
MMAT: Mixed Method Appraisal Tool
MOC: The Mosaic Outpatient Clinic
PRISMA: Preferred reporting items for systematic reviews and meta-analysis
